# Natural Stilbenoids Isolated from Grapevine Exhibiting Inhibitory Effects against HIV-1 Integrase and Eukaryote MOS1 Transposase *In Vitro* Activities

**DOI:** 10.1371/journal.pone.0081184

**Published:** 2013-11-28

**Authors:** Aude Pflieger, Pierre Waffo Teguo, Yorgos Papastamoulis, Stéphane Chaignepain, Frederic Subra, Soundasse Munir, Olivier Delelis, Paul Lesbats, Christina Calmels, Marie-Line Andreola, Jean-Michel Merillon, Corinne Auge-Gouillou, Vincent Parissi

**Affiliations:** 1 Université François Rabelais de Tours, EA 6306, UFR Sciences Pharmaceutiques, Parc Grandmont, Tours, France; 2 Groupe d'Etude des Substances Végétales à Activité Biologique, EA 3675 - UFR Pharmacie, Université Bordeaux Segalen, Institut des Sciences de la Vigne et du Vin (ISVV), Bordeaux, France; 3 Plateforme Protéome - Centre Génomique Fonctionnelle, UMR 5248 CBMN, Université Bordeaux Segalen, Bordeaux France; 4 LBPA, CNRS, Ecole Normale Supérieure-Cachan, France; 5 Cancer Research UK, London Research Institute, Clare Hall Laboratories, Potters Bar, United Kingdom; 6 Laboratoire MFP, UMR 5234-CNRS, Université Bordeaux Segalen, Bordeaux, France; Salute San Raffaele University School of Medicine, Italy

## Abstract

Polynucleotidyl transferases are enzymes involved in several DNA mobility mechanisms in prokaryotes and eukaryotes. Some of them such as retroviral integrases are crucial for pathogenous processes and are therefore good candidates for therapeutic approaches. To identify new therapeutic compounds and new tools for investigating the common functional features of these proteins, we addressed the inhibition properties of natural stilbenoids deriving from resveratrol on two models: the HIV-1 integrase and the eukaryote MOS-1 transposase. Two resveratrol dimers, leachianol F and G, were isolated for the first time in *Vitis* along with fourteen known stilbenoids: *E*-resveratrol, *E*-piceid, *E*-pterostilbene, *E*-piceatannol, (+)-*E-ε-*viniferin, *E-ε-*viniferinglucoside, *E*-scirpusin A, quadragularin A, ampelopsin A, pallidol, *E*-miyabenol C, *E*-vitisin B, hopeaphenol, and isohopeaphenol and were purified from stalks of *Vitis vinifera* (Vitaceae), and moracin M from stem bark of *Milliciaexelsa* (Moraceae). These compounds were tested in *in vitro* and *in vivo* assays reproducing the activity of both enzymes. Several molecules presented significant inhibition on both systems. Some of the molecules were found to be active against both proteins while others were specific for one of the two models. Comparison of the differential effects of the molecules suggested that the compounds could target specific intermediate nucleocomplexes of the reactions. Additionally *E*-pterostilbene was found active on the early lentiviral replication steps in lentiviruses transduced cells. Consequently, in addition to representing new original lead compounds for further modelling of new active agents against HIV-1 integrase, these molecules could be good tools for identifying such reaction intermediates in DNA mobility processes.

## Introduction

DNA mobility is a crucial mechanism involved in genome evolution and in many vital cellular functions such as replication, transcription and the processes of viral and bacterial infection. One of the key parameters in these enzymatic processes is the specificity of DNA recognition. Polynucleotidyl transferases are involved in the cleavage and paste process of a wide variety of DNA products. Interestingly, this family includes several enzymes sharing similar functions (cleavage and strand transfer) but interacting with a variety of different DNA fragments through different mechanisms. These enzymes are known as the DDE/DDD enzymes, according to the amino acid triad involved in the catalytic functions. The nucleophilic substitution reactions are assisted by divalent metal cofactors [1], generally a pair of divalent metal cations (Mg^++^ or Mn^++^) that are thought to be coordinated by three carboxylates of the catalytic core. Both transposases and integrases are DDE/DDD enzymes.

Transposases are proteins that assume the mobility of class II transposable elements, which are DNA elements that move around within their host genome *via* a DNA intermediate. Among them, the *mariner* group consists of well-characterized transposons belonging to the large *ITm* superfamily [2]. Biochemical data accumulated during the past decade from three models (*Mos1* from *D. mauritiana*, *Himar1* from *H. irritans*, and *Hsmar1* from *H. sapiens*) have led to a convergent picture of their transposition cycle [3]. All *mariners* are short elements, of about 1300 base pairs, and are flanked by inverted terminal repeats (ITRs). They transpose using a cut-and-paste mechanism involving several steps. First, sequence-specific binding of the transposase homodimer occurs at one of the ITRs, forming a so-called single-end complex (SEC2). Next, synaptic complex assembly is obtained by the addition of the second ITR to SEC2, giving the paired-end complex (PEC). DNA strands are then cleaved by the transposase, promoting the excision. Once the pre-integration complex (PIC) has been produced, the target DNA is captured, followed by integration of the element into a TA target dinucleotide. The structure of a PIC involving the full-length *Mos1* transposase (MOS1) was recently solved [4] and a model of the MOS1 dimer was proposed [4]. However, full understanding of the *mariner* transposition cycle is far from complete with regard to organization of the complexes, and inhibitors of each step are still required in order to obtain the full picture.

DNA mobility is conserved among the retroviral integrase family as HIV-1 IN. HIV-1 replication requires the stable insertion of the genome under its DNA form catalyzed by the retroviral integrase enzyme (IN, for a recent review on retroviral IN see [5], [6], [7]). This step first involves 3′-processing of the viral DNA ends generated during the reverse transcription reaction, leading to exposure of the 3′ hydroxyl groups of the invariant CA dinucleotides. Next, IN inserts both 3′ ends of the viral DNA into the opposite strands of the chromosome DNA during a concerted integration reaction. Even if the full length HIV-1 IN structure is not yet solved, biochemical studies have revealed several nucleoprotein intermediates required for the integration reaction [8], [9]. The stable synaptic complex, SSC, maintains the two viral ends close to a tetrameric IN structure for their 3′processing maturation. This tetrameric IN bound to the viral DNA constitutes the active intasome. After binding of the target DNA, the strand transfer complex (STC) is formed and engages the processed viral ends for their integration into the host DNA. The recent crystallization of the intasome of another retrovirus, the human prototype foamy virus (PFV), provided many insights into the functional organization of the protein inside these active complexes [10], [11]. Modelling of the HIV-1 intasome based on these structural data makes it possible to depict the HIV-1 integration mechanism [12]. However, the lack of a complete structure of the HIV-1 IN, either as a separate protein in solution or in the context of the functional intasome, limits the rational design of inhibitors against this important replication step.

Owing to the similarities found between transposases and retroviral integrases and the need to find new antiviral compounds, we developed a pharmacological approach to compare our MOS1 and HIV-1 IN models. Resveratrol has been previously demonstrated to be highly reactive in a broad variety of fields including cancer, cell cycle and virology [13], [14], [15]. Additionally, polycyclic compounds like stilbenes have been reported to inhibit polynucleotidyl transferases, such as retroviral integrases and transposases [13], [16]. Thus the strategy was to compare the effect of different stilbenoid compounds purified here for the first time from *Vitis vinifera* grapevine. The compounds were isolated and then tested in specific HIV-1 IN and MOS1 *in vitro* assays. Some of them were found to be active against both proteins and others proved specific for one of the two models, suggesting that they could target different reaction intermediates. Comparison of the differential effects of the molecules found with the two models suggests that these molecules could be new lead compounds for the rational design of anti-HIV-1 IN agents. Additionally, these molecules could be used as tools for identifying the reaction intermediates in DNA mobility systems.

## Materials and Methods

### Chemistry

#### Plant material

Stems of *Vitis vinifera* Merlot cv. were obtained at “Domaine de Merlet”, Pessac-Leognan appellation, in the Bordeaux region, in February 2005, with the agreement of the domain's owner. This study did not involve endangered or protected species.

#### General experimental procedures

Fractionation was performed on a FCPC200® apparatus provided by Kromaton® Technologies (Angers, France) and fitted with a rotor made of 20 circular partition disks (1320 partition cells: 0.130 ml per cell; total column capacity of 204 ml; dead volume: 32.3 ml). The rotor was entirely filled with the aqueous stationary phase in the ascending mode without rotating. For the first CPC step, methyl ter-butyl ether (MTBE) extract was separated by four consecutive CPC runs. An average of 1.8 g was injected in each run. For each run, we dissolved the MTBE extract in 8 ml of the organic/aqueous phase mixture (1∶1).After injection, the organic (upper) mobile phase was pumped into the column in the ascending mode at a flow-rate of 3 ml/min. Then, the rotation speed was increased from 0 to 1000 rpm. Fractions of 9 ml were collected in each tube (3 min/tube). The content of the outgoing organic phase was monitored by an online UV absorbance measurement at λ = 280 nm. Fractions were also analysed by thin-layer chromatography on silica gel pre-coated 60 F_254_ plates (Merck). The plates were developed by spraying with an anisaldehydesulphuric reagent containing 5 ml *p*-anisaldehyde, 90 ml ethanol and 5 ml sulphuric acid, and the compounds were detected at 254 and 366 nm [17]. Some compounds were finally purified from their mixtures obtained by CPC on a Semi-Preparative Varian Prostar Apparatus. A C18 reversed phase column (Prontosil, 250 mm×8.0 mm 5.0 µm, Bischoff) was used for purification. The mobile phase was composed of two solvents: A, 0.025% TFA in water and B, MeCN. The UV absorption was monitored with a Varian 345 dual wavelength UV-Vis detector simultaneously at 280 and 306 nm. The analytical HPLC separations were conducted on a C_18_ column (Prontosil, 250 mm×4.0 mm 5.0 µm, Bischoff) equipped with a guard column of the same nature. The UV-Vis spectra of all compounds were recorded between 200 and 450 nm with a 2 nm step. The analyses were carried out on a Thermo Finnigan Surveyor chromatographic system (Thermo Electron, France).

#### Extraction and Isolation

Dried and finely ground stems of *V vinifera* Merlot *cv*. (3.15 kg) were extracted with 2 times 20 L of acetone/water (6/4, v/v) at room temperature under agitation, twice for 12 hours. After filtration, the aqueous acetone solution was concentrated at 30–35°C under reduced pressure. The residual aqueous phase was successively extracted with *n*-heptaneand methyl *tert*-butyl ether (all solvents were purchased from Scharlau, Sentmenat, Spain). The M*t*BE extract was reduced in vacuum at 30–35°C and lyophilized to yield 44 g of crude extracts mainly composed of stilbenoids.

After testing the “Arizona” [18] solvent system, system K (n-heptane/ethyl acetate/methanol/water 1/2/1/2 v/v) was finally chosen for the first partition of the MTBE extract. After the CPC separation of MTBE extract (7.86 g), 16 fractions were obtained, eight in ascending mode and eight in descending mode.

Fraction 1 (fr. 1–3) mainly containing *E*-resveratrol and (+)-*E*-ε-viniferin was submitted to a second CPC step using the M solvent system (n-heptane/ethyl acetate/methanol/water 5/6/5/6 v/v). *E*-resveratrol could be separated in ascending mode, whereas (+)-*E*-ε-viniferin and *E*-vitisin B were separated in descending mode [17].

Fraction 3 (fr.5) was fractionated with a second CPC step using Arizona solvent system L (n-heptane/ethyl acetate/methanol/water 2/3/2/3 v/v). The major stilbenes, *E*–piceatannol and *E*-scirpusin A, were purified in ascending mode, whereas *E*-miyabenol C was separated in descending and purified by Semi-prep HPLC. The elution program at 3 ml/min was 20% B (0–5 min), 20–26% B (5–10 min), 26% B (10–22 min), 26–50% B (22–52 min), 50% B (52–55 min) and 50–100% B (55–56 min), 100% B (56–64 min), 100–20% B (64–65 min), 20% B (65–72 min).

Pallidol a symmetrical resveratrol dimer and (+)-ampelopsin A were purified from Fraction 8 of the first CPC using the Arizona solvent system J (n-heptane/ethyl acetate/methanol/water 2/5/2/5 v/v) in ascending mode.

(+)-*E*-ε-viniferin glucoside and (+)-*E*-piceid were purified from the descending mode Fraction 11 of the first CPC by semi-prep HPLC. The elution program at 3 ml/min was 15–20% B (0–5 min), 20–26% B (5–13 min), 26% B (26–35 min), 26–50% B (35–75 min), 50% B (75–85 min).

The descending mode fraction 14 arising from the first CPC separation contained a multitude of phenolic compounds, especially stilbenes. The Arizona J system (*n*-heptane/ethyl acetate/methanol/water 2∶5∶2∶5 v/v) proved to be the most adequate for purifying this fraction. 131 tubes were collected, 117 in the ascending mode and 14 in the descending mode, grouped in 10 fractions. From tubes 84–107 of the ascending mode, flavanoids: (+)-catechin, (-)-epicatechin, epicatechin-3-*O*-Gallate, astilbin and stilbenoids: leachianol F and G, quadrangularin A, hopeaphenol and Isohopeaphenol were purified by semi-preparative HPLC. The elution program at 3 ml/min was 5–10% B (0–5 min), 10% B (5–13 min), 10–20% B (13–26 min), 20% B (26–30 min), 20–26% B (30–37 min), 26% B (37–49 min), 26–100% B (49–56 min).

Air-dried and finely powdered bark of *Miliciaexcelsa* (Moraceae), which is commonly known as iroko, was macerated in methanol for 48 h at room temperature, followed by filtration and concentration. This yielded a crude MeOH extract that was washed with hexane and extracted with ethyl acetate. The ethyl acetate extract was separated over a silica gel chromatographic column using a gradient of CH_3_Cl-MeOH as eluent to give Moracin M [19].

#### Structural identification

Further characterization of the compounds was performed on a Thermo Finnigan LCQ Advantage ion-trap spectrometer equipped with an electrospray source (Thermo Electron). The software used for data acquisition and reprocessing was Xcalibur (Thermo Scientific). For HPLC/ESI-MS experiments, we used ultrapure water (resistance <18 MΩ) from an Elga source acidified with 0.025% MS grade trifluoroacetic acid (TFA) (Applied Biosystems) (solvent A) and acetonitrile (solvent B). An HPLC gradient at 1 ml/min used for optimal separation of the compounds contained in all MTBE extracts and further purified fractions was: 15% B (0–5 min), 15% to 20% B (5–13 min), 20% to 26% B (13–26 min), 26% B (26–35 min), 26% to 38% B (35–55 min), 38% to 50% B (56–60 min), 50% to 100% B (60–61 min), 100% B (61–70 min), 100% to 15% B (70–71 min), 15% B (71–80). The HPLC effluent at 1 ml/min was introduced into the electrospray source in a post-column splitting ratio of 9∶1 (100 µl/min). The following MS conditions were used for ionisation, desolvation, focusing and detection: Spray Voltage +4.5 kV, Sheath Gas Flow Rate 23, Auxiliary Gas Flow Rate 3 (ratio sheath gas/auxiliary gas), Heated Capillary Temperature 220°C, Capillary Voltage 26 V, Tube Lens Offset 45 V, Scan Range *m/z* 150–2000. Helium (He) was used as the collision gas and nitrogen (N_2_) as the nebulizing gas. Data were obtained both in positive and negative ionization mode, but most of the compounds and almost all hydroxystilbenic compounds were assigned after their positive mass spectra (higher sensitivity). All isolated compounds were analysed by ^1^H-NMR, ^13^C-NMR and 2D spectroscopy in acetone-d6 and methanol-d4. The spectra were recorded on an AC 300 and Avance DRX 500 Bruker spectrometer (Wissembourg, France).

### HIV-1 IN procedures

#### Protein

HIV-1 IN was purified from a yeast expression system using the IN_Hybrid_ methods described previously [20].

#### *In vitro* Integration assays

Standard concerted integration reactions were performed as described previously [20] with some modifications. Briefly, purified HIV-1 IN (600 nM) was pre-incubated with both the 5′-end-labeled donor DNA (15 ng) containing the unprocessed or processed U3 and U5 LTR sequences and the target DNA plasmid pBSK^+^ (150 ng) at 0°C for 20 min in a total volume of 5 µl. Then the reaction mixture (20 mM HEPES, pH 7.5; 10 mM DTT; 10 mM MgCl_2_; 15% DMSO; 8% PEG, 30 mM NaCl) was added and the reaction proceeded for 120 min at 37°C in a total volume of 10 µL. Incubation was stopped by adding a phenol/isoamyl alcohol/chloroform mix (24/1/25 v/v/v). The aqueous phase was loaded on a vertical 1% agarose gel in the presence of 1% bromophenol blue and 1 mM EDTA. After separation of the products, the gel was treated with 5% TCA for 20 min, dried and autoradiographied. All IN activities were quantified by scanning the bands (half site plus full site integration products) after gel electrophoresis and autoradiography using the Image J software. Both target DNA and donor plasmids were kind gifts from Dr. K Moreau (Université Claude Bernard-Lyon I, France). The target corresponds to the plasmid pBSK^+^ (Stratagene, La Jolla, California) carrying the zeocin resistance-encoding gene. The following reagent was obtained through the NIH AIDS Reagent Program, Division of AIDS, NIAID, NIH: Raltegravir (Cat # 11680) from Merck & Company, Inc.

#### 3′processing assay

The 3′ processing reaction was performed under the same conditions as concerted integration (20 mM HEPES, pH 7.5; 10 mM DTT; 10 mM MgCl_2_; 15% DMSO; 8% PEG, 30 mM NaCl) but for one hour at 37°C and using 1 pmol of 5′ radiolabelled ODN 70/72 (hybrid between ODN 70: 5′GTGTGGAAAATCTCTAGCAGT3′ and ODN 72: 5′ACTGCTAGAGATTTTCCACAC3′). The reaction was stopped by adding 10 µl of loading buffer (95% formamid, 20 mM EDTA, 0.05% bromophenol blue) and heating at 90°C for 5 min. The reaction products were analysed by electrophoresis on 15% polyacrylamide gels with 7 M urea in Tris-borate-EDTA (TBE) pH 7.6 and autoradiographied. All IN activities were quantified by scanning the bands after gel electrophoresis and autoradiography using the NIH software.

### *In vivo* analysis of the lentiviral early replication steps

#### Transduction of 293T cells

293T cells were plated in 48-multiwell plates at 50,000 cells/well using 400 µL of DMEM (Invitrogen, Carlsbad, CA) containing 10% (v/v) foetal calf serum (FCS, Invitrogen) and 50 µg/mL of gentamycin (Invitrogen). Infection was assayed using the pRRLsin-PGK-eGFP-WPRE VSV-G pseudotyped lentiviruses produced as described in [21] after or without treatment with the compounds. After three washed with PBS (140 mM NaCl, 3 mM KCl, 8 mM Na2HPO4, 1.5 mM KH2PO4 pH 7.4) the cells were transduced with lentiviruses (optimized M.O.I  =  10 leading to about 30–40% of transduced cells containing one copy of integrated viral DNA) in a final volume of 400 µl. After ten days cells are washed two times with PBS then treated with trypsin for 5 minutes at room temperature and centrifugated two minutes at 2000 rpm. The pellet is washed two times in PBS. After resuspension in 200 µl PBS/200 mM EDTA, quantification of fluorescence was performed using 10,000 cells on a FACS Calibur (Beckton-Dickinson, San Jose, CA). Plots were analyzed using the FCS express v3.00.0103. Data are presented as the percentage of cells showing a significant level of fluorescence or as the fluorescence intensity calculated by X-median of fluorescence intensity of all cells.

#### Cytotoxicity measurement

The cellular cytotoxicity of the compounds was determined by measuring the cellular survival of cells treated with increasing concentrations of molecules for two days using a standard MTS [3-(4,5-dimethylthiazol-2-yl)-5-(3-carboxymethoxyphenyl)-2-(4-sulfophenyl)-2H-tetrazolium assay (Promega CellTiter 96 AQueous One Solution Cell Proliferation Assay).

#### Quantification of HIV-1 DNA population

Cells were harvested 8 h, 24 h and 48 post-infection by centrifugation of 2.10^6^ to 10.10^6^ aliquots and cell pellets were kept frozen at −80°C until further analysis. Total DNA (including integrated HIV-1 DNA and episomal HIV-1 DNA) was extracted using the QiAmp blood DNA mini kit (Qiagen, Courtaboeuf, France) according to the manufacturer's protocol. Elution was performed in 50 µl of elution buffer. The quantification of the total viral DNA, integrated and 2LTR forms was performed using the conditions and primers described in [22]. The 1LTR circles were quantified using the conditions and primers described in [23]. The total HIV-1 DNA was amplified by quantitative real-time PCR using the light Cycler Instrument (Roche Diagnostics, Meylan, France). Total cell DNA was extracted with a QIAampblood DNA minikit (QIAGEN). Quantifications of total HIV-1 DNA, 2-LTR circles and integrated HIV-1 DNA were performed by quantitative PCR on a LightCycler instrument (Roche Diagnostics) using the fit point method provided in the Light Cycler quantification software, version 4.1 as previously described [24]. Copy number of 2-LTR circles was determined in reference to a standard curve prepared by amplification of quantities ranging from 10 to 1.10^6^ copies of plasmid comprising the HIV_LAI_ 2-LTR junction. Cell equivalents were calculated based on amplification of the β-globin gene (two copies per diploid cell) with commercially available materials (Control Kit DNA; Roche Diagnostics). 2-LTR circles, total and integrated HIV-1 DNA levels were determined as copy numbers per 10^6^cells and 2-LTR circles and integrated DNA were expressed as percentage of total viral DNA.

### MOS1 procedures

#### Protein

The wild type *Mos1* transposase (MOS1: 345 amino acids) was produced and purified as a fusion protein linked to the maltose-binding protein by using the pMal-c2 system (New England Biolabs) and following the manufacturer's instructions.

#### Transposition assays

*In vitro* transposition reactions were performed using pBC-3T3 as both the donor DNA and the target, as previously described [25]. Briefly, the basic transposition reaction mixtures contained 10 mM Tris-HCl (pH 9), 50 mM NaCl, 20 mM MgCl_2_, 0.5 mM EDTA, 5% glycerol, 5 ng/ml BSA, 600 ng pBC-3T3, and 80 nM purified MBP-MOS1 (added last) in a volume of 20 µl. Inhibitors (25 or 10 µM, as specified) or DMSO (2%) were added to the reaction mixtures before the transposase. The reactions were allowed to proceed for 30 min at 30°C. After phenol-chloroform extraction and ethanol precipitation, 10% of the reaction mixture was transformed in electro-competent JM109 *E. coli* (2 mm cuvette, 1.5 kV in a MicroPulser™BioRad). Appropriate dilutions of each reaction mixture were plated on LB-tetracycline (12.5 µg/ml) agar and LB-chloramphenicol (80 µg/ml) agar to score the transposition rate. The transposition rate was the number of Tet^R^ colonies divided by the number of Chloram^R^ plus Tet^R^ colonies. Each condition was done at least three times in independent experiments.

#### Excision assays

Basic excision reaction mixtures contained 10 mM Tris-HCl (pH 9), 50 mM NaCl, 20 mM MgCl_2_, 0.5 mM EDTA, 0.5 mM DTT, 100 ng BSA, 600 ng of super-coiled pBC-3T3, and 80 nM MBP-MOS1 (added last) in a volume of 20 µl. Molecules (25 or 10 µM, as specified) or DMSO (2%) were added to the reaction mixtures before the transposase. The reactions were allowed to proceed at 30°C for 30 min before 2 µl of stop solution (loading buffer) were added. The reaction mixtures were then incubated at 65°C for 10 min. Each reaction was analysed by overnight electrophoresis at 2.7 V/cm on a TAE-buffered 1% agarose gel with ethidium bromide (0.3 mg/ml). After electrophoresis, the gel was photographed on a transilluminator. ImageGauge 4.22 software (Fujifilm) was used to quantify the bands. Each condition was tested at least twice.

#### Integration assays

These assays were performed using pre-cleaved 3′ITRs (PC-ITR), *i.e.* ITRs in the configuration expected after excision. Double-strand ITRs were obtained by annealing equimolar amounts of 70-NTS-PN (5′GGTGTACAAGTATGAAATGTCGTTTGATCCCCCGGGCTGCAGG3′) and 70-TS-PC (5′AATTGCTGCAGCCCGGGGGATCAAACGACATTTCATACTTGTACACCTGA3′) single-strand oligonucleotides. Prior to annealing, the transferred strand (70-TS-PC) was 5′-end labelled using T4 polynucleotide kinase (Promega) and γ-^32^P-ATP following the manufacturer's instructions. Oligonucleotides were provided by Eurofins MWG Biotech (Germany) or Eurogentec (Belgium). Reaction mixtures containing 10 mM Tris-HCl (pH 9), 50 mM NaCl, 1 mM DTT, 5% glycerol, 100 ng BSA, 100 nM of labelled PC-ITR and 100 nM MBP-MOS1 in a final volume of 20 µl were incubated at 30°C for 30 min. The pBC plasmid (which contained the *cat* gene, a hot-spot for *Mos1* insertion) was then added to 30 ng/µL along with 5 mM MgCl_2_. Molecules (25 or 10 µM, as specified) or DMSO (2%) were added to the reaction mixtures along with the target DNA to estimate their effect on integration. Reactions were carried out for 3 hours at 30°C. 0.1 mg/ml proteinase K and 0.1% SDS were added to stop the reaction. DNA products were purified by phenol-chloroform extraction and ethanol precipitated using standard techniques. DNA products were resuspended in DNA loading buffer, then separated onto a 1% agarose gel in 1X TAE (Tris Acetate EDTA) buffer. The gel was dried onto a nitrocellulose membrane (Hybond N+, GE Healthcare), scanned using a Storm, and analysed with ImageQuant software. Signals were quantified using ImageQuant software. Efficiency of integration was calculated as the intensity of the integration signal divided by the intensity of free target signal plus integration signal. Each condition was tested at least twice.

## Results

### Isolation of natural stilbenes

**Figure 1:**
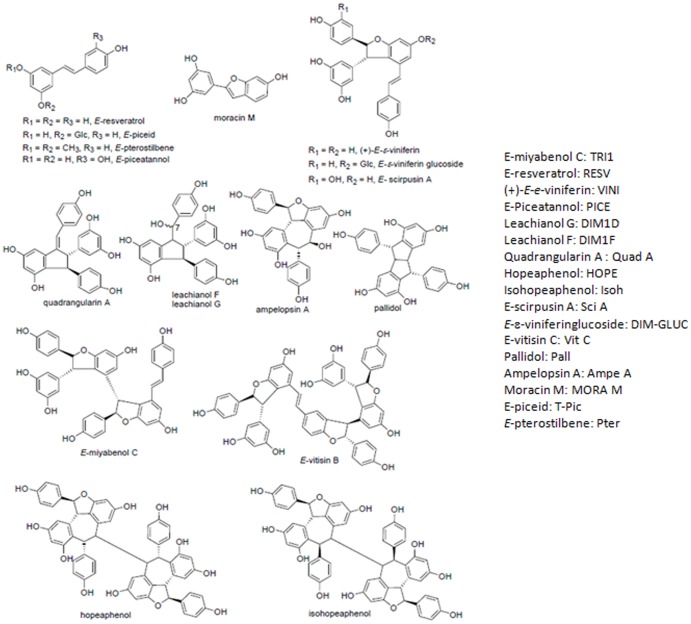
General structure of resveratrol derivative stilbenes purified form *Vitis vinifera* grapevine. The compounds are divided into three families: monomers, dimers and higher order oligomers of the resveratrol motif. A list of the acronyms used in this work is reported in the figure.

The chemical structures of stilbenoids isolated and characterized from grapevine and *Millicia excelsa* are shown in **figure 1**. The primary objectives of the study were to investigate the isolation of new lead compounds deriving from resveratrol and to evaluate their *in vitro* activity in HIV-1 IN and MOS-1 transposase inhibitory assays for further modelling of new agents active against HIV-1 integrase.

The extraction procedure is described in the materials and methods section. Fractionation of crude stilbenoid extract was performed by centrifugal partition chromatography (CPC) using adapted Arizona solvent systems [18]. CPC is a type of counter-current chromatography. It is a very versatile separation technique that does not require a solid stationary phase. It relies simply on the partition of a sample between the two phases of an immiscible solvent system.

Purification of the stilbenoids from CPC fractions by semi-preparative HPLC was guided by electrospray ionisation mass spectrometry (ESI-MS). Following the fractionation and purification procedure, 15 stilbenes were isolated and characterized by one- and two-dimensional NMR spectroscopy. Two stilbene dimers, leachianol F and G (see NMR data in **Table S1**), were isolated for the first time in grapevine. These compounds seem to be potent topoisomerase II inhibitors and were isolated for the first time as a natural product in *Sophoraleachiana* roots [26]. Besides these two dimers, four monomers (*E-*resveratrol, *E*-piceid, *E*-pterostilbene, and *E*-piceatannol), six dimers ((+)-*E*-ε-viniferin, *E*-ε-viniferinglucoside, E-scirpusin A, ampelopsin A, quadrangularin A, Pallidol), a trimer (E-miyabenol C) and three tetramers (hopeaphenol, isohopeaphenol, vitisinD) were isolated. All the compounds were solubilized in DMSO at concentrations ranging from 1 to 20 mM and stored at −20°C in darkness. After identification, all the purified molecules were tested on HIV-1 integrase and MOS1 transposase *in vitro* activity.

### Selection of natural stilbenes active on HIV-1 *in vitro* integration

**Figure 2:**
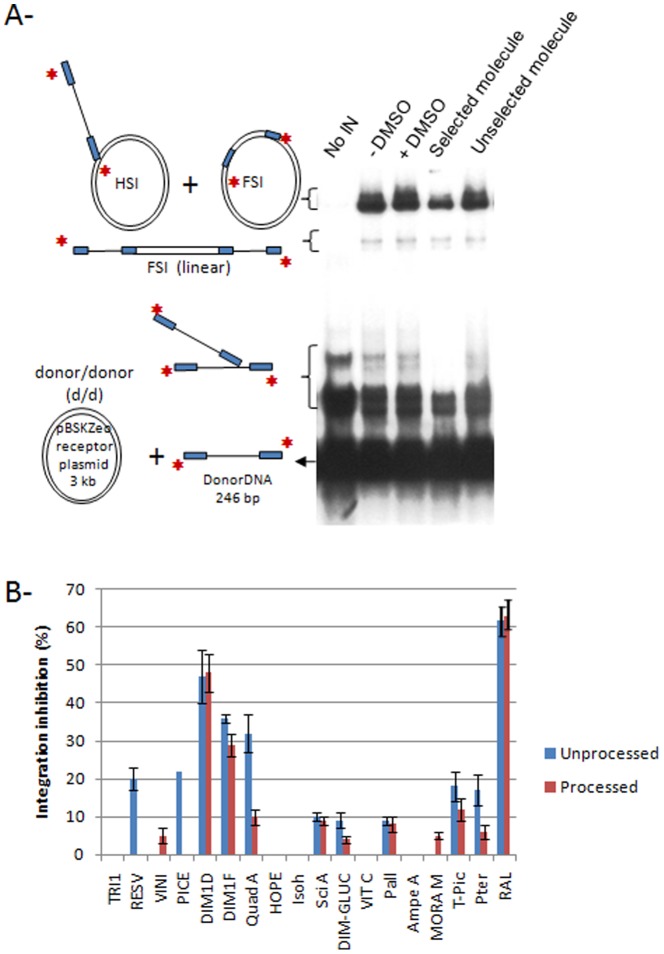
Selection of stilbenes inhibiting *in vitro* HIV-1 integration. Concerted integration assay was performed without IN or using 600 µM of compounds. The reaction products were loaded onto 1% agarose gel. The position and the structure of the different products obtained after half-site (HSI), full-site (FSI) and donor/donor integration (d/d) are shown in **A** as well as the integration profile obtained in presence of the DMSO control, selected (here DIM1D) or unselected molecule (here TRI1). The integration products were quantified as reported in materials and methods and the integration inhibition is shown in **B** using the integration activity detected in presence of DMSO as reference. All quantifications are shown as the means from at least three independent experiments ± standard deviation (error bars). We also reported the inhibitory effect observed with 10 µM raltegravir (RAL).

To identify compounds with anti-IN activity, we tested them in a typical global concerted integration assay using a recombinant HIV-1 IN enzyme as reported before [20]. A first round of selection was performed by testing the isolated molecules on concerted integration reaction at a final 10 µM concentration, using either the 246 bp donor containing either the pre-processed HIV-1 U5 and U3 viral ends (reaction reproducing the strand transfer activity) or the blunt natural HIV-1 U5 and U3 unprocessed ends (reaction reproducing both the 3′processing and the strand transfer reactions, see materials and methods section for the accurate description of the substrates and the assay). **Figure 2A** shows a typical concerted integration assay and the profile obtained with a selected inhibitory molecule (leachianol G) or an inactive compound (*E*-miyabenol C). The effects of the molecules on *in vitro* integration activity were compared to the reaction observed in presence of the DMSO control (see lane 3 of **figure 2A**).

As shown in **figure 2B**, 7 of the 17 molecules tested were found inactive or exhibited only a slight effect (< to 5% of inhibition) in both conditions (using the unprocessed or the processed substrate): *E*-miyabenol C, (+)-*E*-ε-viniferin, hopeaphenol, isohopeaphenol, vitisin B, moracin M and ampelopsin A. These molecules were excluded from further analyses in the HIV-1 IN systems. The other molecules were then analysed with increasing concentrations in concerted integration assays and compared to the well characterized raltegravir (RAL) anti-integrase compound.

**Figure 3:**
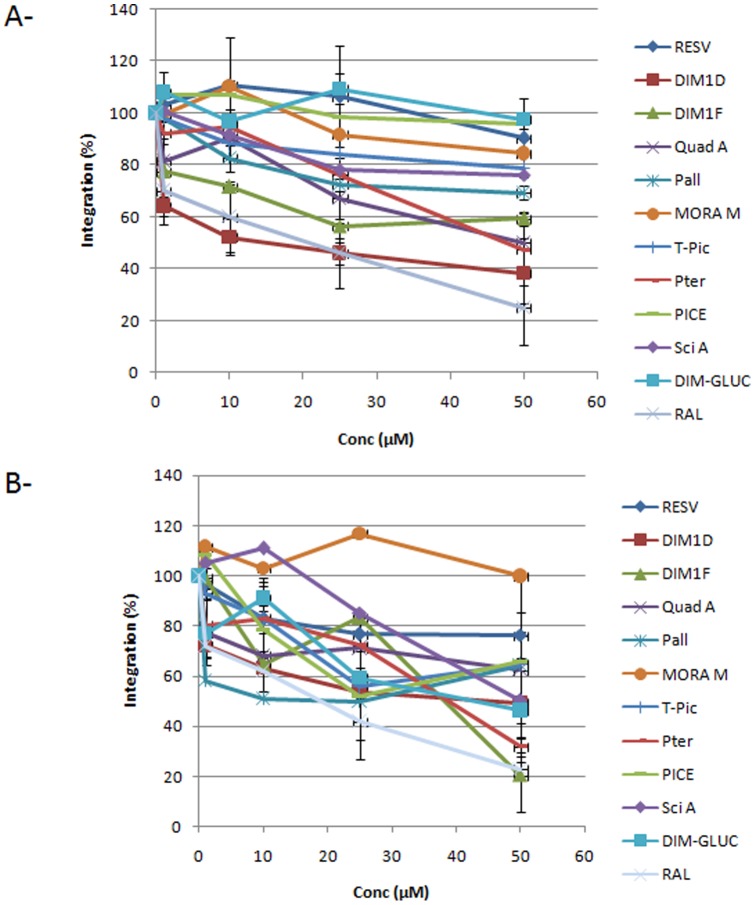
Effect of stilbene compounds on *in vitro* HIV-1 concerted integration. Concerted integration assays were performed using 600(**A**) or unprocessed (**B**) donor DNA incubated with increasing concentration of compounds. The integration products were quantified as reported in materials and methods section and the percentage of integration is shown as the means from at least three independent experiments ± standard deviation (error bars). We also reported the inhibitory effect observed with raltegravir (RAL).

The data in **figures 3A and 3B** confirm the first selection since leachianol G, leachianol F, quadrangularin A, *E*-scirpusin A, pallidol, E-piceid and E-pterostilbene inhibited both activities with various efficiencies (the best inhibition was observed with leachianol G and leachianol F, reaching 30–60% inhibition at 50 µM). Interestingly, some molecules exhibited specificities for one reaction over the other. For example, *E*-ε-viniferin glucoside, *E*-resveratrol and *E*-piceatannol were found to inhibit integration from the unprocessed substrate without having any detectable effect on the processed DNA, and quadrangularin A was a better inhibitor of integration from unprocessed than from processed DNA. The raltegravir molecule was always found better inhibitor than the selected compounds. However under our conditions some molecules showed inhibitory efficiencies close to raltegravir (leachianol G, leachianol F and E-pterostilbene). Since these results suggested a differential effect of the molecules on the 3′processing steps compared to the strand transfer reaction monitored when using the pre-processed donor, we analysed the molecules in an independent 3′processing assay.

**Figure 4:**
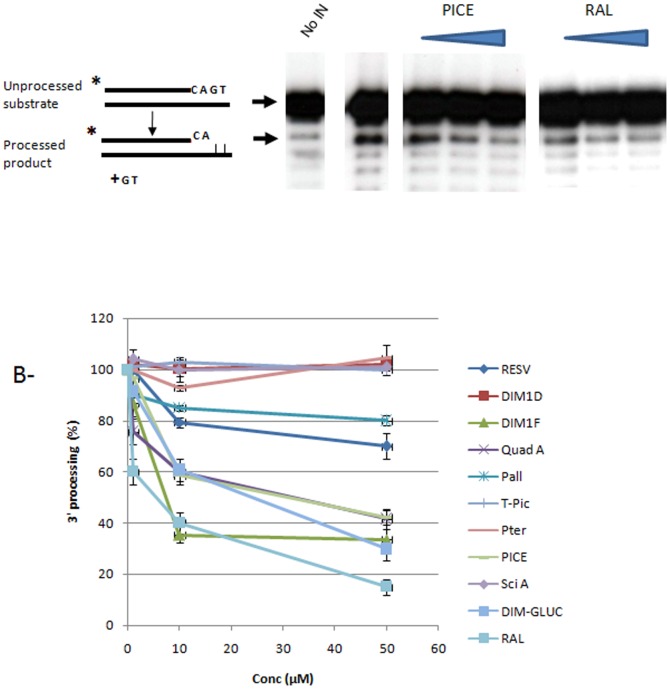
Effect of stilbene compounds on *in vitro* 3′processing reaction catalysed by HIV-1 integrase. Standard 3′processing assay was performed as reported in materials and methods section using 600 nM IN, 1 pmol of 5′ radiolabelled 21 bp ODN 70/72 mimicking the viral U5 end and increasing concentration of molecules. An example of 3′ processing profile obtained with a selected inhibitory compound (here *E*-piceatannol (PICE) and the raltegravir (RAL) control molecule is shown in **A** as well as the position and the structure of the expected 19 bp reaction product. The 19 bp processed product was quantified as reported in materials and methods section and the percentage of 3′ processing is shown in **B** as the average from at least three independent experiments ± standard deviation (error bars).

As shown in **figure 4** **,** only: *E*-ε-viniferinglucoside, leachianol F, *E*-piceatannol quadrangularin A and *E*-resveratrol showed an inhibitory effect on 3′processing, thereby confirming the data in **figure 3**. The low 3′processing inhibitory effect found for *E*-scirpusin A and leachianol G compared to their inhibition on concerted integration in the presence of the processed donor suggests that these compounds could be specific to the target capture and/or strand transfer reaction. In contrast, the 3′processing inhibition found for *E*-ε-viniferin glucoside, *E*-resveratrol and *E*-piceatannol compared to their poor effect on the strand transfer reaction reported in **figure 3A** suggests that they could be more active on the maturation step of the viral end. Again the RAL molecule showed a better 3′processing inhibition property than the selected compounds.

### Effect of the selected molecules on the early steps of lentiviral replication

**Figure 5:**
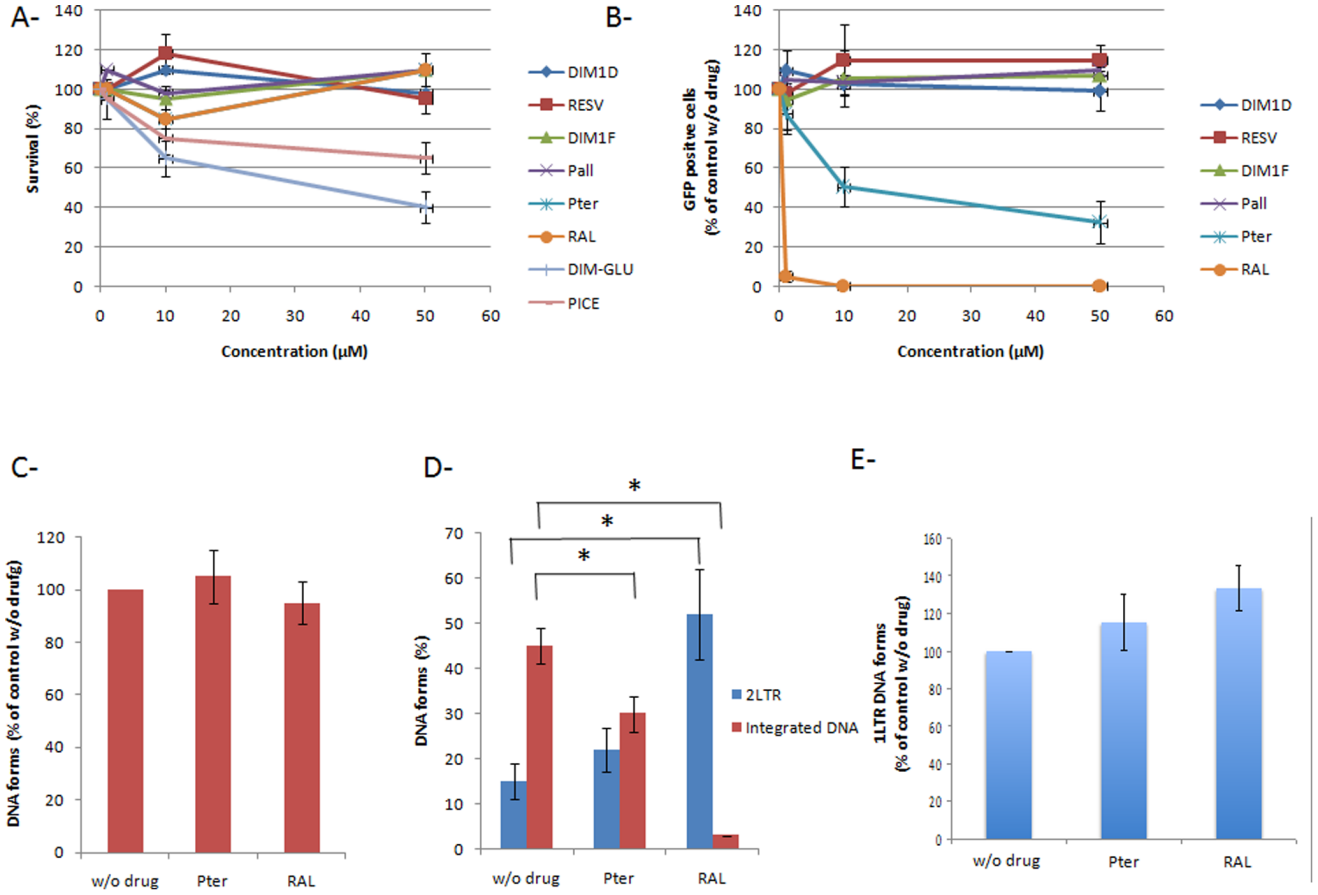
Effect of selected molecules on the early steps of VSV-G pseudotyped lentiviruses replication in 293T cells. After 24materials and methods (**A**). The cell survival is reported as a percentage of control without molecules and as the means from at least three independent experiments ± standard deviation (error bars). The effect on replication was measured by FACS quantification of the GFP expressed by the cells transduced with VSV-G pseudotyped lentiviruses (**B**). Results are reported as a percentage of GFP positive cells as the average from at least three independent experiments ± standard deviation. Total (**C**), Integrated, 2LTR (**D**) and 1LTR (**E**) circles viral DNA were quantified (8 h post-infection of the total DNA and 24 h post-infection for the integrated and 2LTR circle DNA) by quantitative PCR. An example of quantification obtained with an inhibitory compound (here Pter) is shown. Total viral DNA is reported as percentage of control without drug. Integrated and 2LTR circles DNA are reported as a percentage of each form comparing to total viral DNA. Results are reported as the average from at least three independent experiments ± standard deviation, *p<0.05.

In order to determine whether some molecules could be active on the retroviral integration step in a cellular context we tested the compounds in a typical single round infectivity assay. The best molecules selected *in vitro* were first tested for their cytotoxicity in 293T cells by a 72 hours treatment with increasing concentration (1–50 µM) and by measuring the cell survival in a standard MTS (3-[4,5-dimethylthiazol-2-yl]-2,5- diphenyltetrazoliumbromide) assay. As reported in **figure 5A, ***E*-piceatannol and *E*-ε-viniferinglucoside showed some toxicity and, thus, were excluded from further analyses in this work. 293T cells were then treated with the remaining molecules for 24 hours and transduced with VSV-G pseudotyped lentiviruses carrying a GFP encoding gene (standard optimized M.O.I. = 10). Since the GFP expression is directly linked to integration efficiency integration was evaluated by quantifying the GFP fluorescence by flux cytometry ten days after transduction in order to avoid any contamination with GFP expression from plasmids used for lentiviruses production. As reported in **figure 5B** only the E-pterostilbene was found to induce an integration inhibition.In order to determine whether viral DNA synthesis was also affected by the compoundthe total viral DNA wasquantified as reported in materials and methods section for an efficient 10 µM concentration of E-pterostilbene. No significant change was detected in the total DNA amounts after 8 hours post-transduction (**figure 5C**) nor 24 h post-transduction (data not shown) indicating thatthe E-pterostilbene does not affect reverse transcription. To better confirm an effect at the integration step integrated DNA and 2LTR circles were also quantified 24 h post-transduction. We observed a slight but reproducible decrease in the integrated DNA amounts (**figure 5D**). Interestingly non significant increase in the 2LTR circles DNA was detected suggesting that the E-pterostilbene treatment could lead to an unusual inhibition of the integration step which remain to be further determined. However since the 2LTR/integrated DNA ratio is not clearly indicative of an integration impairment especially when the decrease in integrated DNA amount is low additional steps could be affected by the molecule as nuclear import. This has been investigated by measuring the 1LTR circles mainly formed by circularisation of linear viral DNA in the nucleus [23]. The quantification demonstrated that no decrease in the 1LTR circles amount was found whatever the condition studied (**figure 5E**) indicating that the nuclear import was not significantly impaired by the E-pterostilbene compound and confirming an effect during the integration process.

### Selection of natural stilbenes active on MOS1 *in vitro* transposition

**Figure 6:**
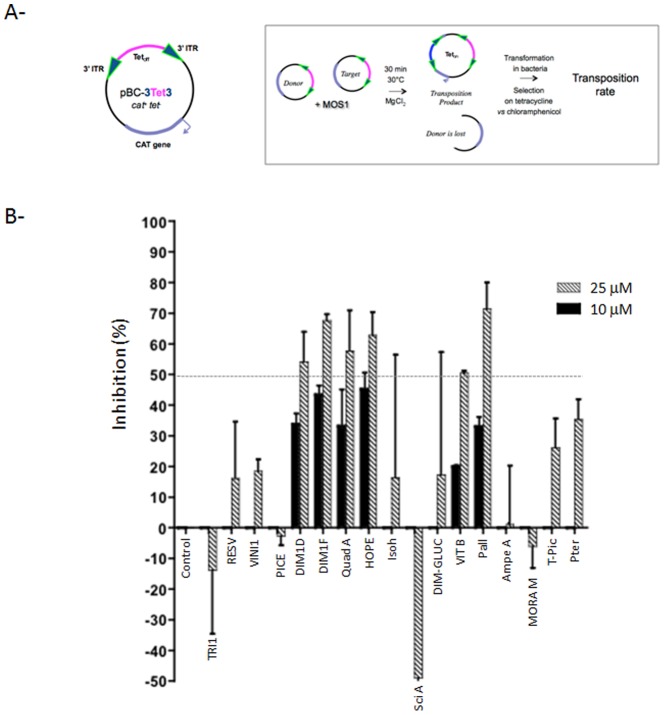
Selection by *in vitro* transposition of natural stilbene compounds active against *Mos1* transposase. **(A)** *in vitro* transposition test principle. pBC-3T3 was used both as the donor of pseudo-*Mos1* and as the target for integration. It contained the pBR322 tetracycline resistance gene (without promoter) flanked by two *Mos1* 3′ITR. This plasmid was unable to confer resistance to *Escherichia coli* cells in tetracycline concentrations over 10 µg/ml. Upon transposition, MOS1 excises the pseudo-*Mos1* from one pBC-3T3 molecule, and then triggers its reinsertion within the *cat* gene of another pBC-3T3 molecule [28]. Transposition events are revealed by promoter tagging, the tetracycline resistance being activated through the *cat* gene promoter. Transposition rates were quantified as reported in materials and methods section and the transposition inhibition is shown in **(B)** using the transposition rate obtained in presence of DMSO as reference. Values are the means from at least two independent experiments ± standard deviation (error bars). Only compounds giving a transposition rate inhibition over 50% at 25 µM were assayed at 10 µM.

To identify the natural stilbenes active on MOS1, the 17 molecules were tested using an *in vitro* genetic “hop” experiment, which is the most sensitive test available as yet. Purified MOS1 was incubated with the pBC-3T3 plasmid, which was used both as the transposon donor and the target plasmid. This plasmid contains the pBR322 tetracycline resistance gene (without promoter) framed by two *Mos1* ITRs. This reconstitutes a pseudo-*Mos1* element named 3T3. We took advantage of the fact that the *cat* gene (present in the pBC backbone) is a hotspot for *Mos1* integration [27]. Consequently, transposition events are revealed by promoter tagging, the tetracycline resistance being activated through the *cat* gene promoter (**figure 6A**). Transposition events were recovered by bacterial transformation with selection for tetracycline resistance, as a gain-of-function landmark for transposition. A first round of selection was performed by testing the molecules at a final concentration of 25 µM. Controls correspond to assays performed without molecule but with DMSO, which is routinely used as the solvent. As shown in **figure 6B**, leachianol G, leachianol F and quadrangularin A, which were previously shown to be active against HIV-1 IN, also induced over 50% inhibition of the whole MOS1 transposition activity. In addition, three other compounds were shown to be active against MOS1: hopeaphenol, *E*-vitisin B and pallidol. To confirm the efficiency of the six molecules, they were similarly assayed at 10 µM (**figure 6B**). Since they appeared to be still active at 10 µM, they were further characterized in order to identify the targeted step of the transposition cycle.

**Figure 7:**
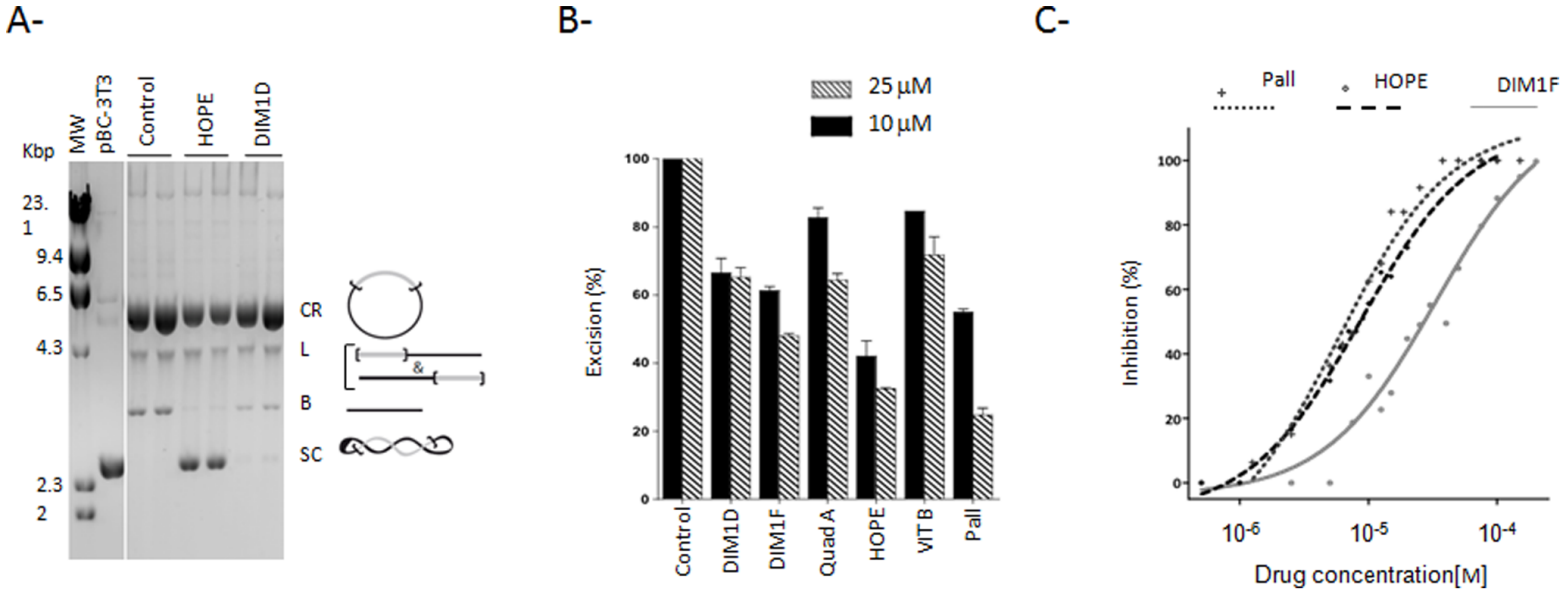
Effect of the selected molecules on *Mos1* excision. **(A)** *In vitro* excision assays were performed using a super coiled plasmid pBC-3T3 and purified MBP-MOS1. The resulting products were loaded onto agarose gel. Data obtained with two molecules (25 µM of DIM1F or HOPE) or DMSO (control) are shown. The pBC-3T3 plasmid used as the substrate is shown in lane2.Molecular weight markers are indicated on the left. The various products are depicted on the right and their positions on the gel are indicated. SC: super coiled donor. First strand nicking at one transposon end generates an open circular product (OC). Second strand nicking linearizes the donor (L), yielding the single-end break product. A similar sequence of nicks at the other transposon end yields the double-end break products, which consist of the plasmid backbone (B) plus the excised transposon fragment (not shown). **(B)** DNA products detected in the gel were quantified, and the appearance of the backbone (B) was used as a measure of excision. The control assay (DMSO) was taken as the reference (100% excision) and data obtained for each molecule (at 25 and 10 µM) are expressed as excision relative percentage. Each point was repeated at least twice. **(C)** IC_50_ were determined for three molecules (Pall, HOPE, and DIM1F) using a three log range of compound concentrations. The percentage inhibition of excision was plotted as a function of the log of compound concentrations. Experimental data were fitted on a sigmoid dose-response curve using Prism software.

The ability of the selected molecules to inhibit excision was tested *in vitro* using the pBC-3T3 plasmid as the transposon donor. MOS1 triggers the excision of the pseudo-*Mos1* from the plasmid producing two linear DNA fragments, the transposon and the plasmid backbone, together with intermediate products (open circle plasmids which contained single strand nicks and linear plasmids which contained a double strand nick at one ITR). After excision, the transposon could be reintegrated, making it difficult to detect in electrophoresis. The activity of the molecules was measured by quantifying the plasmid backbone, the end product of the reaction. The assays were performed with final concentrations of 25 and 10 µM molecules. Controls correspond to assays performed without molecule but with DMSO. **Figure 7A** shows profiles recovered from a typical excision test with molecules more or less inhibiting this step. Reactions were performed at least twice for each selected molecules (leachianol G, leachianol F, quadrangularin A, hopeaphenol, vitisin B and pallidol) and the excision activity is reported in **figure 7B**. Hopeaphenol, pallidol and leachianol F inhibit significantly the *in vitro* excision reaction over 50% at 25 µM. Their IC_50_ were determined using concentrations range and found to be 8.5 µM for Hopeaphenol, 8 µM for pallidol and 30 µM for leachianol F (**figure 7C**). The six selected molecules were then tested for the integration step of *Mos1* transposition.

**Figure 8:**
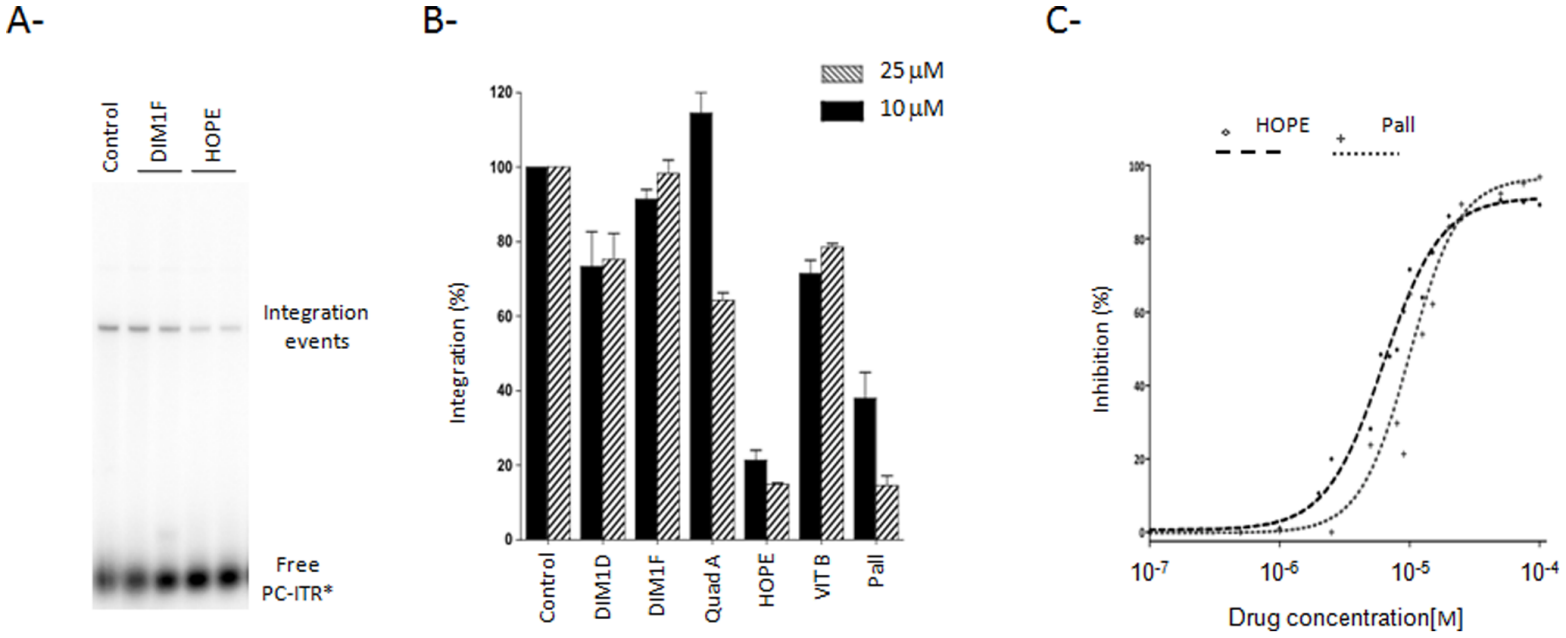
Effect of molecules on *Mos1* ITR integration. **(A)** *In vitro* integration assays were performed using labelled PC-ITR and purified MBP-MOS1. pBC was used as the target. The resulting products were loaded onto agarose gel and detected using a Storm. Data obtained with two molecules (25 µM of DIM1F or HOPE) or DMSO (control) are shown. The various products are indicated on the right. Each point was repeated at least twice. **(B)** DNA products detected in the gel were quantified and the appearance of labelled plasmid (integration products) was used as a measure of integration. The control assay (DMSO) was taken as the reference (100% integration) and data obtained for each molecule (at 25 and 10 µM) are shown as relative percentage of integration. **(C)** IC_50_ were determined for two molecules (HOPE, and DIM1F) using a four log range of compound concentrations. The percentage inhibition of integration was plotted as a function of the log of compound concentrations. Experimental data were fitted on a sigmoid dose-response curve using Prism software.

The assay used for this step is similar to the HIV-1 IN concerted integration. Briefly, pre-cleaved labelled ITRs are used to form the integration complex. A super-coiled plasmid is used as target DNA. Integration events give a linear labelled plasmid, which can be detected. In these tests, the molecules are added after formation of MOS1-ITR complexes, along with a target plasmid to solely examine the integration inhibition. **Figure 8A** shows a typical integration test and the profiles obtained in presence or not of molecules. Controls correspond to assays performed without molecule but with DMSO. Reactions were performed at least twice for each selected molecule (leachianol G, leachianol F, quadrangularin A, hopeaphenol, vitisin B and pallidol) and the excision activity is shown in **figure 8B**. Under these conditions, hopeaphenol and pallidol significantly inhibited the integration step. Their IC_50_ were determined using a concentration range and found to be around 6.5 µM for hopeaphenol, and 10 µM for pallidol (**figure 8C**).

## Discussion

Raltegravir, the only drug targeting the integration step in the HIV-1 life cycle, has given β-ketoenol integrase (IN) strand transfer inhibitor (STI) pride of place in treating HIV-1 infection. However, the emergence of viral strains resistant to β-ketoenol STI requires the continued search for novel IN inhibitors interfering with HIV-1 IN in a mechanistically different manner. Retroviral INs share common features with polynucleotidyl transferases like MOS1 transposase. By comparing these enzymes pharmacologically, it is possible to identify new compounds acting on these DNA mobility enzymes and to obtain new information about the mechanism they catalyse, thereby helping in the rational design of new antiviral compounds.

Polyphenols, which are among the most developed IN inhibitors, exhibit a different mechanism of action compared with β-ketoenol STI. Some of them exhibit strong IN inhibitory activity and anti-viral activity at nanomolar range). Such compounds are undoubtedly attractive candidates for future inhibitor design, as they should be effective against STI-resistant viral strains and display a synergistic effect when combined with the current STI. Consequently, we focused our work on new natural polyphenol molecules isolated from the *Vitis vinifera* grapevine.

In addition to the isolation of several stilbene compounds, we report here the first purification of two resveratrol dimers in *Vitis vinifera*:leachianol G and leachianol F. To our knowledge, this is the first description of such natural compounds in grapevine.

The assay of the compounds in *in vitro* HIV-1 integration tests showed that some compounds efficiently inhibited one or all reactions catalysed by IN. Interestingly, some compounds like leachianol G, *E*-piceid and *E*-pterostilbene were more active on strand transfer reaction than 3′processing, suggesting a mechanism of action close to that of STI. In contrast, we also found molecules with a greater effect on 3′reaction than on integration like *E*-resveratrol and *E*-ε-viniferin glucoside. Comparison between the effects of the *E*-resveratrol reference molecule and those of its derivatives sheds light on the chemical basis of the inhibition. Indeed, although the inhibitory property of *E*-resveratrol is low, active point modifications of the chemical backbone largely increase the reactivity of the compounds against HIV-1 IN. The glycosylation or methylation found in *E*-piceid and *E*-pterostilbene led to an increase in their ability to inhibit integration. This suggests the importance of these positions in the inhibition process and indicates that they are good candidates for further chemical modifications in order to optimize their inhibition efficiency.

More strikingly, the comparison between leachianol G and leachianol F suggests that the change in the stereochemistry in C7 of these hydroxylated dimers also changes their inhibitory potential. Leachianol G was found to be more active on strand transfer than on 3′processing in contrast to leachianol F, which was active on both reactions. This suggests that such modifications of resveratrol dimers could direct the inhibitory property of the molecule from one reaction to the other. Interestingly the best compounds selected in this work as leachianol G and *E*-pterostilbene have shown *in vitro* integration inhibition efficiency close to raltegravir. Cellular assays confirmed that the *E*-pterostilbene molecule was able to inhibit the early steps of a VSV-G pseudotyped lentivirus but without reaching the raltegravir efficiency. Quantification of the viral DNA forms suggests that the compound could inhibit with a low efficiency the integration of viral DNA without affecting reverse transcription nor nuclear entry of the viral DNA but. Interestingly no significant effect was observed on the 2LTR circles amount suggesting that the E-pterostilbene could lead to an unusual inhibition of the retroviral integration. One hypothesis could be an inhibition at the post-integration DNA repair (PIR) step. Indeed an inhibition of the integration locus repair leading to an elimination of the integrated DNA could also lead to similar phenotype. Thus, in order to further optimize antiviral strategy using this molecule or derivates, the mechanism of integration inhibition in cell remains to be further determined as well as the intracellular entry property of the compounds and their possible secondary targets in the cellular context.

All molecules were also tested in *in vitro* assays reproducing integration and excision reactions catalysed by MOS1. The most active compounds against HIV-1 IN, pallidol, quadrangularin A, leachianol G and leachianol F, were also active against MOS1 activities. This suggests that these resveratrol dimers could be highly reactive and do not target a specific functional complex for HIV-1 or MOS1 enzymes. In contrast, *E*-resveratrol and *E*-ε-viniferin glucoside, which act more specifically on HIV-1 3′processing reaction, and *E*-piceid and *E*-pterostilbene, which is more efficient on the integration reaction, were not active on MOS1. This suggests that they target specifically functional HIV-1 intermediate complexes.

Hopeaphenol, was found to be selectively active on MOS1, with an IC_50_ between 6-8 µM. Interestingly, the stereoisomer isohopeaphenol was not active, suggesting the requirement of a specific stereoisomer to achieve inhibition. These molecules complete a first series of MOS1 inhibitors recently described as inhibiting the integration step with an IC_50_ of about 1 µM [27]. The effectiveness of the natural products described herein, as well as the products previously described remains to be improved.

In conclusion, we report stilbenoid compounds active on *in vitro* HIV-1 IN activities that could serve as lead compounds for further development of new optimized molecules. For this purpose, a rational design would be based on the difference in inhibition efficiency between chemicals with regard to their structure. The differences found between HIV-1 IN and MOS1 against the selected compounds indicate that the models involve different nucleocomplex intermediates of the reaction, which highlights differences in the entire integration process. Hopeaphenol, which inhibited MOS1 without having any detectable effect on HIV-1 IN, and *E*-resveratrol, *E*-ε-viniferin glucoside, *E*-pterostilbene and *E*-piceid, which acted more specifically on HIV-1 IN activities, could serve as tools to increase our understanding of the reactions catalysed by each enzyme after identification of the functional complexes targeted by the molecules.

## Supporting Information

Table S1 NMR data on the two stilbene dimers, leachianol F and G. After purification the compounds were analyzed by ^1^H-NMR, ^13^C-NMR and 2D spectroscopy in acetone-d6 and methanol-d4. The spectra were recorded on an AC 300 and Avance DRX 500 Bruker spectrometer (Wissembourg, France).
(DOCX)
