# Supplementary material for: Natural Stilbenoids Isolated from Grapevine Exhibiting Inhibitory Effects against HIV-1 Integrase and Eukaryote MOS1 Transposase In Vitro Activities
Source: PLoS One. 2013 Nov 28;8(11):e81184. doi: 10.1371/journal.pone.0081184 (PMC3842960; doi:10.1371/journal.pone.0081184)
Supplement: Table S1 — NMR data on the two stilbene dimers, leachianol F and G. After purification the compounds were analyzed by 1H-NMR, 13C-NMR and 2D spectroscopy in acetone-d6 and methanol-d4. The spectra were recorded on an AC 300 and Avance DRX 500 Bruker spectrometer (Wissembourg, France). (DOCX) [file pone.0081184.s001.docx]

| N° | leachianol F | | leachianol G | |
| --- | --- | --- | --- | --- |
|  | δ_C_ | δ_H_ (J Hz) | δ_C_ | δ_H_ (J Hz) |
| 1 | 136.2 |  | 135.8 |  |
| 2(6) | 128.8 | 6.84 (*d*, 8.6) | 129.5 | 7.07 (*d*, 8.5) |
| 3(5) | 115.3 | 6.66 ( *d*, 8.6) | 115.6 | 6.73 (*d*, 8.5) |
| 4 | 157 |  | 157.3 |  |
| 7 | 76.5 | 4.44 (*d*, 8) | 77.2 | 4.47 (*d* 7.8) |
| 8 | 61.8 | 3.34 (*dd*, 3.9, 8.1) | 62.6 | 3.34 (*dd*, 7.8, 3.2) |
| 9 | 148.7 |  | 147.4 |  |
| 10 | 122.4 |  | 123.2 |  |
| 11 | 154.9 |  | 154.8 |  |
| 12 | 102.2 | 6.29 (*d*, 2.3) | 102.3 | 6.22 (*d*, 1.6) |
| 13 | 158.7 |  | 158.6 |  |
| 14 | 106.1 | 6.56 ( *d*, 2.3) | 105.6 | 5.73 (*d*, 1.6) |
| 1’ | 137.4 |  | 138.0 |  |
| 2’(6’) | 129.3 | 6.83 (*d* 8.6) | 129.4 | 6.88 (*d* 8.5) |
| 3’(5’) | 115.5 | 6.71 (*d* 8.6) | 115.2 | 6.72 (*d* 8.5) |
| 4’ | 156.2 |  | 156.3 |  |
| 7’ | 55.6 | 4.21 (*d*, 4.2) | 56.1 | 4.27 (*d*, 3.2) |
| 8’ | 59.4 | 2.93 (*t*, 4.2) | 59.1 | 3.50 (*t*, 3.2) |
| 9’ | 150.6 |  | 151.5 |  |
| 10’(14’) | 105.9 | 5.90 (*d*, 2.3) | 106.2 | 6.15 *brd* |
| 11’(13’) | 159.9 |  | 159.3 |  |
| 12’ | 101 | 6.11 (*t*,2.3) | 101.0 | 6.16 *brt* |

Table S1 : 1H, and 13C NMR data of leachianols F and G in acetone-*d*6
